# Digital neuromorphic technology: current and future prospects

**DOI:** 10.1093/nsr/nwad283

**Published:** 2023-11-03

**Authors:** Steve Furber

**Affiliations:** Department of Computer Science, The University of Manchester, UK

## Abstract

Digital approaches to brain-inspired computing have advanced apace over recent years - where is the state-of-the-art and what does the future hold?

Neuromorphic technology has diversified considerably from its origins in the seminal work by Carver Mead and his group at Caltech in the 1980s [1]. That early work focussed on the analogy between the equations describing the flow of ions in biological neurons and the equations describing the flow of carriers in field-effect transistors operating in the subthreshold region. Though research continues into both subthreshold and above-threshold analogue neuromorphic circuits, this is complemented by developments in digital neuromorphic systems that take inspiration from biological neuronal systems at higher levels in their architectures, solving the low-level equations with digital hardware or software rather than by direct circuit-level emulation.

Digital neuromorphic technology has a number of advantages over its analogue counterpart, notable among which are its ability to access the most advanced technology nodes and the consistency and repeatability of its behaviour. Against this, analogue neuromorphic technology has intrinsic advantages in terms of energy-efficiency, though this may be offset by the difficulties in implementing analogue circuits on the most advanced technology nodes. Analogue circuits are also closer to emulating biology. Both approaches have the potential to benefit from innovations in device technology such as using memristors as adaptive synapses.

Current digital neuromorphic systems include the Intel Loihi [2] and Loihi 2, and the University of Manchester SpiNNaker [3] (shortly to be upgraded to SpiNNaker2). Both hardware platforms are capable and robust, and widely used by the neuromorphic research community. Other notable examples include the IBM TrueNorth and Tianjic [4] from Tsinghua University in China.

What is less developed is our understanding of how to configure these systems for useful applications, and high-level tools to support the development of those applications. PyNN [5] is a tool that enables spiking neural networks to be described at the level of populations of neurons and projections that connect one population to another; this is a similar level to that of a register-transfer level description of computer hardware, and is appropriate for describing biological neural networks, but does not offer the levels of abstraction available in mainstream AI to describe convolutional and deep networks. If neuromorphic technology is to find widespread adoption by the engineering community, for example to address the need for energy-efficient inference in Internet of Things (IoT) edge devices, then more functional ways of describing spiking neural networks will be required. One tool that does offer functional descriptions of spiking networks is Nengo [6], and this has proved very productive for developing both neurorobotic control systems and partial brain models such as Spaun [7].

While tools and software frameworks for spiking neural networks and neuromorphic platforms are lagging behind those available for conventional deep and convolutional artificial neural networks, there has been considerable progress in exploring the potential application space for neuromorphic systems. Much of this work has been led by Intel's Neuromorphic Computing Lab and the Intel Neuromorphic Research Community (INRC) [8]. The results show considerable promise for neuromorphic technologies in application domains such as event-based sensing and closed-loop control for robotics.

The focus of the SpiNNaker work has been much more towards brain science where, in a friendly competition with high-performance computers and GPUs, the neuromorphic platform was the first to achieve biological real-time performance on a model of the cortical microcircuit [9]. Several other brain regions have also been modelled on SpiNNaker, including the cerebellum [10]. The cerebellum is particularly challenging for neuromorphic systems as its Purkinje cells have in the region of 100 000 input connections (synapses) from other neurons. The model described here does not quite reach this degree of fan-in, though on average the Purkinje cells in the model have 28 000 synapses.

SpiNNaker2 [3], the second-generation SpiNNaker chip, delivers more than an order of magnitude improvement in functional density and energy efficiency over the first-generation chip, and offers support for hybrid neural systems (as does Tianjic [4]), combining spiking neural networks with conventional artificial neural networks. It has been designed to deliver high performance in single-chip edge AI applications and in massively parallel multi-million core data centre configurations.

Future prospects for digital neuromorphic technology depend very much on progress being made in our understanding of how best to represent data in spiking networks, what learning rules are effective in what contexts, how to exploit sparse representations and sparse connectivity, and how to put all of these aspects together into effective systems. Brain science will likely have a lot of influence on how all of this progresses, though this is still impeded by a basic lack of understanding of the fundamental principles of operation of the brain as an information processing system.

In summary, digital neuromorphic hardware technology is good to go, and although the understanding of how to make best use of the hardware still lags behind, significant progress is being made. The challenge remains to understand how to raise the level of abstraction at which spiking networks can be described to something like that achieved in mainstream AI by tools for deep and convolutional networks. The prospect of the widespread adoption of neuromorphic technologies for energy-efficient edge AI and even sparse event-based large language models looms ever closer.
